# Equipping the American Joint Committee on Cancer Staging for Resectable Pancreatic Ductal Adenocarcinoma with Tumor Grade: A Novel Staging System

**DOI:** 10.1155/2020/9093729

**Published:** 2020-09-21

**Authors:** Hu Ren, Chao-Rui Wu, Guo-Tong Qiu, Li-Peng Zhang, Saderbieke Aimaiti, Cheng-Feng Wang

**Affiliations:** ^1^Department of Pancreatic and Gastric Surgery, National Cancer Center/National Clinical Research Center for Cancer/Cancer Hospital, Chinese Academy of Medical Sciences and Peking Union Medical College, Beijing 100021, China; ^2^State Key Lab of Molecular Oncology, National Cancer Center/National Clinical Research Center for Cancer/Cancer Hospital, Chinese Academy of Medical Sciences and Peking Union Medical College, Beijing 100021, China

## Abstract

**Background:**

The 8th American Joint Committee on Cancer (AJCC) staging system for pancreatic ductal adenocarcinoma (PDAC) outperforms its previous version in reproducibility but not in survival discrimination. Tumor grade, an indicator of the aggressive biology of PDAC, has been suggested as a reliable prognostic factor. This study aimed to construct a novel staging system with greater prognostication for resectable PDAC by incorporating tumor grade into the 8th AJCC system.

**Methods:**

A total of 9966 patients with resectable PDAC from the Surveillance Epidemiology and End Results (SEER) database were randomly separated into training and interval validation sets. Another 324 patients from our center were included as an external validation set. We proposed a novel staging system by sorting the substages yielded by a combination of *T*, *N*, and tumor grade based on their overall survival (OS) and grouping them into several stages. Prognostic homogeneity and discrimination were determined using the likelihood ratio *χ*^2^ and the linear trend *χ*^2^ test, respectively. Prognostic accuracies were evaluated by the area under the receiver operating characteristics curve (AUC).

**Results:**

Using the 8th AJCC system, the prognosis of patients within the same stage was quite heterogeneous among different substages. The multivariate Cox model identified the tumor grade (hazard ratio 1.333, 95% confidence interval 1.250–1.423, *p* < 0.001) was an independent prognostic factor of the OS. In the training set, the AUC, homogeneity, and discriminatory ability were superior for the novel staging system than for the 8th AJCC system (0.642 vs. 0.615, 403.4 vs. 248.6, and 335.1 vs. 218.0, respectively). Similar results were observed in the internal and external validation sets.

**Conclusions:**

The novel staging system incorporating tumor grade into the 8th AJCC system was associated with better prognostic accuracy, homogeneity, and discriminatory ability among resectable PDAC patients. Moreover, the novel staging system also allowed possibly adjuvant chemotherapy decisions.

## 1. Introduction

Pancreatic ductal adenocarcinoma (PDAC) remains one of the most lethal human malignancies, with little improvement in survival over the past decades [1]. The five-year survival rate of PDAC is extremely low (approximately 8%), and PDAC is projected to be the second leading cause of cancer mortality in the next decade [2, 3].

The American Joint Committee on Cancer (AJCC) staging system, which includes the depth of tumor invasion (*T*) and nodal status (*N*), was the most frequently used prognosticator for PDAC patients. The 8th AJCC staging system has introduced major revisions to its previous version, including changes to *T* and *N* definitions [4]. The new *T*1–*T*3 definitions completely depend on tumor size, and *T*4 refers to tumors that involved the celiac axis, the superior mesenteric artery, and/or common hepatic artery. Additionally, node-positive disease (previous *N*1) was further categorized into *N*1 (1–3 positive nodes) and *N*2 (≥4 positive nodes). However, although the reproducibility of the 8th AJCC staging system was superior, it did not outperform previous versions in terms of survival discrimination [5, 6]. Survival curves of resectable stages IB, IIA, or IIB disease are not well-separated.

With the evolving understanding of PDAC biology, the spectrum of biologic factors that are predictive of prognosis and treatment response was rapidly expanding [7, 8]. In addition to tumor size and lymph node status, those emerging biologic factors should also be incorporated into the existing staging system for more accurate survival prediction [9–12]. Tumor grade, which represents the aggressive biology of the tumor itself, has been widely confirmed as a prognostic factor in PDAC [13]. Wasif et al. [14] proposed a new staging system by combining the tumor grade and the 7th AJCC stage, which significantly optimized survival discrimination. However, this proposed staging scheme arbitrarily upstaged patients with high tumor grade and downstaged patients with low tumor grade, which was methodologically questionable. Chen et al. [15] have combined tumor grade and the 8th AJCC staging system into a novel staging system using the SEER database. Liu et al. [16] also proposed a novel one by integrating the tumor grade and postoperative CA19-9 levels with the 8th AJCC staging system in their single-institutional dataset. However, neither of them had attempted to cross-validate their staging schemes.

This study aimed to assess the performance of the 8th AJCC staging system and the impact of tumor grade on overall survival (OS). We sought to incorporate tumor grade into the 8th AJCC system to form a novel staging system and validate it both internally and externally.

## 2. Materials and Methods

### 2.1. Study Cohort

The National Cancer Institute's Surveillance, Epidemiology, and End Results (SEER) database, which collects cancer incidence, treatment, and survival data from 18 population-based cancer registries in the US, was used to establish the novel staging system for resectable PDAC. We identified patients with locally resectable PDAC (ICD-O-3 codes 8500/3 and 8140/3, respectively) from 2004 to 2015. Cases with locally unresectable tumor (PDAC with unreconstructable SMV or PV occlusion, and classified as T4 by the 8th AJCC staging system), distant metastasis, history of prior malignancy, age at diagnosis younger than 18 years, and missing information regarding tumor grade, tumor size, tumor extent, and nodal status or OS were excluded. Finally, a total of 9966 patients with resectable PDAC were eligible, which were randomly partitioned into disjoint datasets for model training (*n* = 4983) and internal validation (*n* = 4983). To validate the novel staging system, 324 patients with resectable PDAC that matched the inclusion criteria at the China National Cancer Center between January 2010 and October 2017 were included as an external validation set.

### 2.2. Data Collection

Demographic and clinicopathological variables extracted for each patient were as follows: age at diagnosis, year of diagnosis, gender, tumor size, tumor extent, tumor location (head, body, tail, and others), tumor grade (well-differentiated, moderately differentiated, poorly differentiated, and undifferentiated), nodal status (number of examined lymph nodes and number of positive lymph nodes), the 7th AJCC stage, and survival months. With staging information inferred from the 7th AJCC stage, patients enrolled in the current study were restaged in compliance with new *T*, *N* definitions introduced to the 8th AJCC staging system. Tumor differentiation refers to the extent to which a tumor cell morphologically and functionally resembles a normal cell from the same tissue. The extent of glandular differentiation dictates the histologic grade of PDAC. A tumor with unrecognizable tissue of origin was graded as undifferentiated, < 50% of its components being gland as poorly differentiated, 50%–95% as moderately differentiated, and > 95% as well-differentiated. In this study, to minimize interinstitution and interobserver variance in the pathological assessment of tumor grade, tumor grade was dichotomously subdivided into the low grade (well to moderately differentiated, G1) and the high grade (poorly to undifferentiated, G2).

### 2.3. Statistical Analysis

OS, the primary endpoint in this study, was defined as the duration from the date of initial diagnosis to the date of death or last follow-up (updated on June 1, 2019). The univariate and multivariate Cox proportional hazard regression analysis was performed to identify the independent prognostic predictors for the OS. The OS was analyzed by the Kaplan–Meier survival curves, and log-rank tests were utilized to evaluate the staging systems. The performance of these staging systems was graded based on the area under the curve (AUC), homogeneity, and discriminatory ability. The AUC was compared between the two staging systems using DeLong's method. Homogeneity, a measure of differences in OS among patients with the same stage within each staging system, was calculated using the likelihood ratio *χ*2 through the Cox regression model [17]. Discriminatory ability, a measure of differences in OS among patients in different stages within each staging system, was calculated using the linear trend *χ*^2^ test [18]. All tests were 2-sided, and *p* < 0.05 was considered statistically significant. Statistical analyses were performed using SPSS software version 25.0 (SPSS Inc., Chicago, IL, USA).

## 3. Results and Discussion

### 3.1. Baseline Characteristics

A total of 9966 patients with resectable PADC from the SEER database (2004–2015) were enrolled, which was split equally into a training set (*n* = 4983) and an internal validation set (*n* = 4983). In addition, a total of 324 patients with resectable PDAC diagnosed from 2010 to 2017 at the China National Cancer Center were enrolled as an external validation set. The detailed patients' characteristics of the three cohorts are shown in Table 1. All clinicopathological characteristics were comparable between the training set and the internal validation set. Compared with the training set and internal validation set, the external validation set was associated with a higher proportion of female patients, patients with age at diagnosis <65 years, tumors located at the body and tail of pancreas, higher tumor grade, less than 15 harvested lymph nodes, tumor located within pancreas, less advanced pathological N stage, less advanced 7th AJCC, and less advanced 8th AJCC stage. The median OS of the training set, internal validation set, and external validation set were 19.0, 19.0, and 23.6 months, respectively.

### 3.2. Independent Prognostic Factors of Survival in the Training Set

The univariate and multivariate analysis results are shown in Table 2. In the training set, age at diagnosis (*p* < 0.001), year of diagnosis (*p* < 0.001), tumor grade (*p* < 0.001), number of examined lymph nodes (*p* < 0.001), extrapancreatic invasion (*p* < 0.001), tumor size (*p* < 0.001), and number of metastatic lymph nodes (*p* < 0.001) were identified as independent prognostic factors for OS.

### 3.3. Development of a Novel Staging System in the Training Set

**Figure 1:**
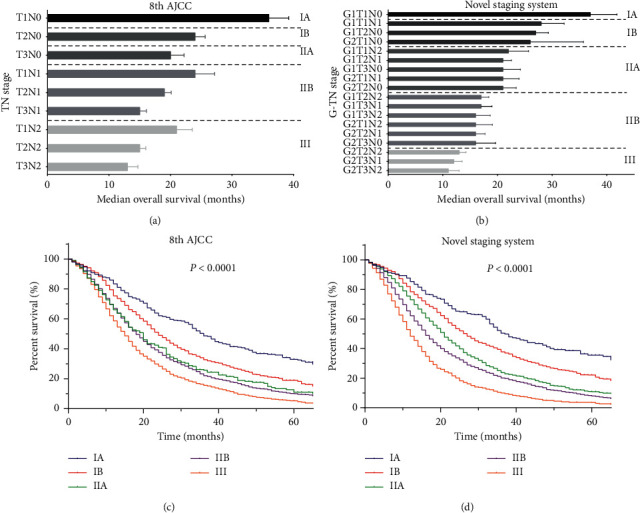
Staging system and corresponding Kaplan-Meier survival curves for the training set; TN stages and survival duration using the 8th AJCC staging system (a), G (grade)-TN stages and survival duration using the novel staging system (b), Kaplan-Meier survival curves for the patients using the 8th AJCC staging system (c), or novel staging system (d).

To evaluate the accuracy and appropriateness of the 8th AJCC staging system, the median OS of each substage from the training set was calculated. Even within the same tumor stage, the median OS was quite heterogeneous across different substages (Figure 1(a)). For example, within stage ?, the median OS for T1N2M0, T2N2M0, and T3N2M0 was 18.5, 14.0, and 11.3 months, respectively (*p* < 0.001). Moreover, patients with stage IIB T1N1M0 tumors survived longer than patients with stage IIA T3N0M0 tumors (median OS: 20.8 vs 17.8 months; *p* < 0.001). Similarly, patients with stage III T1N2M0 tumors survived longer than patients with stage IIB T3N1M0 tumors (median OS: 18.5 vs 13.9 months; *p* < 0.001). Based on the results of multivariate analysis, the tumor grade was incorporated into the 8th AJCC system to form a novel staging system, which resulted in 18 substages. According to the median OS of each substage, these substages were regrouped into five stage groups, including novel IA (G1T1N0), novel IB (G1T1N1, G1T2N0, and G2T1N0), novel IIA (G1T1N2, G1T2N1, G1T3N0, G2T1N1, and G2T2N0), novel IIB (G1T2N2, G1T3N1, G1T3N2, G2T1N2, G2T2N1, and G2T3N0), and novel III (G2T2N2, G2T3N1, and G2T3N2) (Figure 1(b)).

### 3.4. Comparison between the 8th AJCC and the Novel Staging System

**Figure 2:**
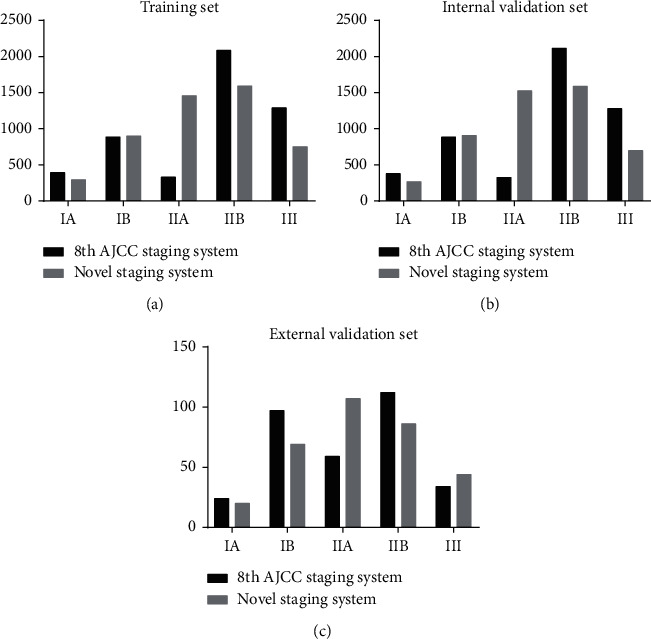
The histogram showing the distribution of patients across different substages within the 8th AJCC or novel staging system.

A histogram showing the distribution of patients across different substages within in the three cohorts are shown (Figure 2). In the training set, 7.9%, 17.8%, 6.6%, 41.8%, and 25.9% of the patients were assigned to stage IA, IB, IIA, IIB, and III (T1-3N2M0) tumors by the 8th AJCC staging system, respectively, while 5.9%, 29.8%, 48.8%, 31.9%, and 15.1% of the patients were assigned to stages IA, IB, IIA, IIB, and III (T1-3N2M0) tumors by the novel staging system, respectively. As to the external validation set, 7.4%, 29.8%, 18.1%, 34.4%, and 10.4% of the patients were assigned to stages IA, IB, IIA, IIB, and III (T1-3N2M0) tumors by the 8th AJCC system, respectively, while 6.1%, 21.2%, 32.8%, 26.4%, and 13.5% of the patients were assigned to stages IA, IB, IIA, IIB, and III (T1-3N2M0) tumors by the novel system, respectively. The proportion of stage IIA disease by the novel stage was much higher than that of stage IIA disease by the 8th AJCC system in the three cohorts.

**Figure 3:**
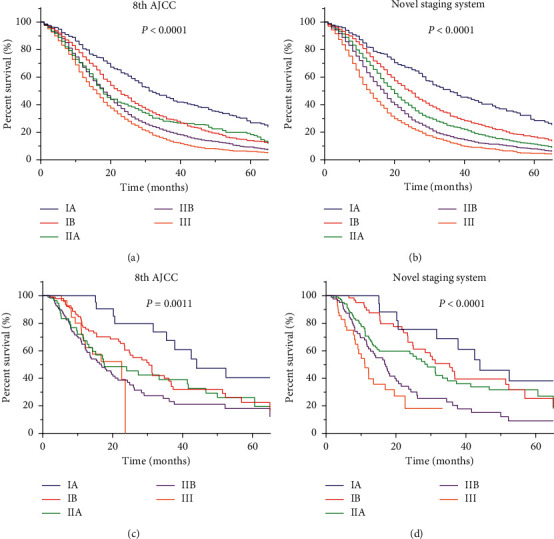
Kaplan-Meier survival curves for the internal validation set using the 8th AJCC staging system (a) or novel staging system (b). Kaplan-Meier survival curves for the external validation set using the 8th AJCC staging system (c) or novel staging system (d).

Suggested by the well-separated survival curves for the three cohorts (Figure 1(d), Figures 3(b) and 3(d)), the novel staging system showed better prognostic discrimination compared with the 8th AJCC staging system (Figure 1(c) and Figures 3(a) and 3(c)). A good separation of survival curves between stages IIA and IIB disease was also noticed. The results of performance evaluation for the three staging systems are shown in Table 3. In the training set, the respective 3-year AUCs of the 8th AJCC and the novel staging system were 0.615, and 0.642, respectively (*p* < 0.001). The likelihood ratios *χ*^2^, which was a measure of homogeneity, of the 8th AJCC and the novel staging system was 248.6 and 403.4, respectively. The discriminatory ability of each system from the linear trend *χ*^2^ test was 218.0 and 335.1, respectively. Similar results were observed in the internal and external validation sets.

### 3.5. This Novel Staging System Predicted Survival Benefits from Adjuvant Chemotherapy

**Figure 4:**
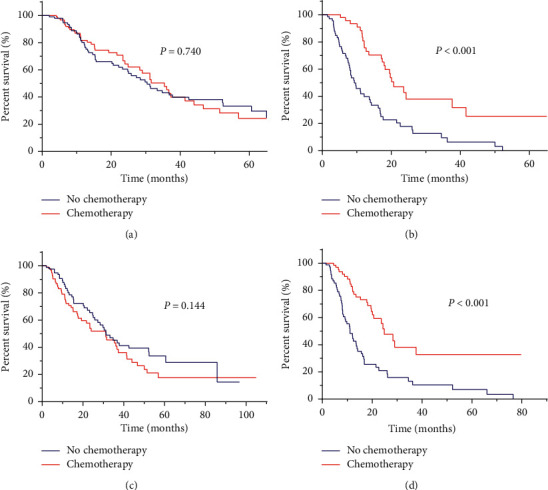
Kaplan-Meier curves showed the response to chemotherapy classified by the novel staging system (stages IA-IIA (a) and stages IIB-III (b)), and the 8th AJCC staging system (stages IA-IIA (c) and stage IIB-III (d)) in the external validation set. Survival curves of the training set and internal validation set are not shown as there was a lack of chemotherapy information in the SEER database.

Analysis of the external validation set showed that the median OS was prolonged by adjuvant chemotherapy (31.2 versus 16.8 months, *p*=0.005). The adjuvant chemotherapy benefit was subsequently determined in each stage within the 8th AJCC and the novel staging system. In the subgroup of patients with novel stage IA- IIA disease, there was no significant difference in median OS between patients who received adjuvant chemotherapy and patients who did not (35.4 versus 30.4 months, *p*=0.740, Figure 4(a)). Patients with 8th AJCC stage IA- IIA disease had a similar result (31.2 versus 31.4, *p*=0.144, Figure 4(c)). Whereas in the subgroup of patients with the novel or 8th AJCC stage IIB-III disease, there was a survival benefit in patients who received adjuvant chemotherapy (novel stage IIB-III, median OS, 20.7 versus 9.3 months, *p* < 0.001, Figure 4(b); 8th AJCC stage IIB-III, median OS, 24.9 versus 11.1 months, *p* < 0.001, Figure 4(d)).

## 4. Discussion

Supported by the previous studies [5, 6, 19, 20], a moderate improvement in the accuracy of prediction was yielded by the 8th AJCC staging system. Nevertheless, the survival curves for stages IIA and IIB disease were not clearly separated in the 8th AJCC system, and the overall discrimination was only marginally enhanced compared with the 7th AJCC system. Thus, the 8th AJCC system for resectable PDAC is far from accurate, and improvement should be considered. So far, revisions of the AJCC staging system for PDAC largely relied on changes to *T* and *N* definitions [4], leading us to consider that a staging system that merely revised the existing parameters might fail to gain a satisfying performance of survival discrimination.

In the novel staging system, a moderate improvement in survival discrimination was demonstrated by the elevated AUCs of survival prediction. A good separation of survival curves between stages IIA and IIB disease was also noticed. Prognostic homogeneity and discriminatory ability were also significantly optimized by the novel staging system. Furthermore, we found that this novel staging system had another role in addition to the prediction of survival for resectable PDAC. Although chemotherapy remained the only effective option of systemic therapy, the survival benefit it offered was quite limited, and only a few patients would respond to chemotherapy [21]. So far, there is a paucity of tools to select the subset of patients who would benefit from adjuvant chemotherapy [22]. In the current study, only patients with the 8th AJCC and novel stages IIB or III disease could benefit from adjuvant chemotherapy, which suggested that the tumor grade as well as the tumor stage were reliable indicators of the tumor burden. As shown in Figure 1, the novel staging system can provide more accurate survival prediction for resectable PDAC by incorporating tumor grade into the 8th AJCC system. The information on tumor grade could inform the efficacy of adjuvant chemotherapy for patients within the same 8th AJCC staging system. We think the novel staging system may be a simple and feasible supplement for the 8th AJCC system to identify patients who would benefit from adjuvant chemotherapy.

Chen et al. [15] have combined tumor grade and the 8th AJCC staging system into a novel staging system using the SEER database. Liu et al. [16] also proposed a novel one by integrating the tumor grade and postoperative CA19-9 levels with the 8th AJCC staging system in their single-institutional dataset. However, the external validations of their staging schemes were lacking in their studies. In contrast, the newly proposed staging system described in this study has been cross-validated in both the SEER database and our center. Furthermore, lots of nomograms in which diverse prognostic factors were included have been developed to yield absolute prediction [10–12]. However, both patients and clinicians were reluctant to use these nomograms, mostly because of their complexity and the difficulty of using them. In contrast, our proposed staging system only included three components and was subdivided into five stage groups, which was simpler than nomograms. The tumor grade was easy to obtain, which was routinely reported by pathologists. Although the incorporation of the tumor grade complicated the staging system, it significantly optimized the prediction accuracy of the existing AJCC staging system.

Most researchers performed an initial analysis in their single-institution dataset before seeking validation within a larger multi-institution dataset, hoping the analysis can be equally effective in other centers [6, 19, 23], while in the current study, we utilized the SEER database as the training set rather than our institution's patients. The sample of our single-institution cohort was not adequate enough to establish a reliable staging system. The sample size of the SEER database was adequate while it only included patients from the USA. Moreover, the characteristics were significantly different between the SEER database and the external cohort, which was possibly reflective of the difference between the western and eastern PDAC patients. Therefore, we decided to establish a novel staging system from the SEER database and validate it in an external cohort to evaluate its applicability in eastern patients.

In the current study, we observed that the nodal status was less advanced in the external dataset, but there was no significant difference in tumor size between the SEER data and the external dataset. A smaller proportion of patients with a tumor located at the head of pancreas in the external dataset may account for the smaller number of harvested and metastatic lymph nodes. Furthermore, the interinstitutional variance also accounts for the discrepancy in part.

This novel staging system tends to downstage node-positive disease (T1N1, T2N1, T1N2, and T2N2) regardless of grade classification. A recent study by Shi et al. [19] demonstrated that the median OS of patients in the same 8th AJCC stage varied widely among the different substages, and they proposed a modified staging system by regrouping the substages. In their proposed staging system, T1N1, T2N1, T1N2, and T2N2 were downstaged as a result of longer survival duration than other substages within the same stage, which was in accordance with our results.

Some limitations should not be ignored. First, despite a superior performance of the novel staging system, the addition of tumor grade created a more complicated system than the AJCC 8th system. Second, the period of the current study was more limited as the preoperative radiographic assessment and postoperative adjuvant therapies of the included patients would be more uniform. Third, we cannot eliminate interinstitution and interobserver variance in protocols for pathological assessment of tumor grade. The two-tiered grading system adopted in this study which divided the grade into high and low could partially minimize that variance. Fourthly, the external validation set has a significantly higher OS compared with the two other sets from the SEER database, which may undermine the efficacy of validation. Finally, the survival discrimination was only moderately improved by the proposed staging system, which owns to insufficient inclusion of predictive factors needed for absolute prediction. For example, although the prognostic value of various clinicopathological factors, such as preoperative CA19-9 level, resection margin status, lymphovascular invasion, nerve invasion, and adjuvant chemotherapy have already been validated by previous studies [16, 21], they were not included as a lack of these information in SEER database.

## 5. Conclusions

In conclusion, our newly proposed staging system was associated with better prognostic accuracy, homogeneity, and discriminatory ability among PDAC patients after pancreatectomy compared with the 8th AJCC staging system. Moreover, the novel staging system also allows possible adjuvant chemotherapy decisions. We believe that the superiority of this staging system stems from new revisions to the 8th AJCC *T* and *N* definitions and the inclusion of tumor grade that reflects the aggressive behavior of PDAC. However, external validation from other populations is necessary.

## Figures and Tables

**Table 1:** Baseline clinicopathologic characteristics.

Characteristics	Training set (*n* = 4983) (%)	Internal validation set (*n* = 4983) (%)	External validation set (*n* = 324) (%)
Age, yrs			
<65	2172 (43.6)	2185 (43.8)	212 (65.4)
≥65	2811 (56.4)	2798 (56.2)	112 (34.6)

Sex			
Male	2461 (49.4)	2478 (49.7)	136 (42.0)
Female	2522 (50.6)	2505 (50.3)	188 (58.0)

Location			
Head	3832 (76.8)	3842 (77.1)	161 (49.7)
Body and tail	704 (14.2)	717 (14.4)	163 (50.3)
Other	447 (9.0)	424 (8.5)	0 (0)

Year of diagnosis			
2004–2009	2049 (41.1)	2010 (40.3)	—
2010–2015	2934 (58.9)	2973 (59.7)	—

Grade			
Low grade	3063 (61.5)	3115 (62.5)	165 (50.9)
High grade	1920 (38.5)	1868 (37.5)	159 (49.1)

Examined lymph nodes			
<15	2187 (46.1)	2221 (46.6)	183 (56.5)
≥15	2558 (53.9)	2547 (53.4)	141 (43.5)

7th AJCC stage			
IA	188 (3.8)	192 (3.9)	7 (2.2)
IB	270 (5.4)	292 (5.9)	36 (11.1)
IIA	1105 (22.2)	1081 (21.7)	130 (40.1)
IIB	3418 (68.6)	3417 (68.5)	151 (46.6)

8th *T* stage			
*T*1	833 (16.7)	801 (16.1)	44 (13.5)
*T*2	2928 (58.8)	3023 (60.7)	179 (54.9)
*T*3	1222 (24.5)	1159 (23.3)	103 (31.6)

8th *N* stage			
*N*0	1610 (32.3)	1591 (31.9)	180 (55.2)
*N*1	2084 (41.8)	2114 (42.4)	112 (34.4)
*N*2	1289 (25.9)	1278 (25.6)	34 (10.4)

Extrapancreatic invasion 8th AJCC stage	4106 (82.4)	4100 (82.3)	250 (76.7)
IA	393 (7.9)	379 (7.6)	24 (7.4)
IB	886 (17.8)	885 (17.8)	97 (29.9)
IIA	331 (6.6)	327 (6.6)	58 (17.9)
IIB	2084 (41.8)	2114 (42.4)	111 (34.3)
III	1289 (25.9)	1278 (25.6)	34 (10.5)

Median OS (95% CI)	19 (18.4–19.6)	19.0 (18.4–19.6)	23.6 (19.4–27.8)

Values in parentheses are percentages.

**Table 2:** Univariate and multivariate analysis for overall survival of patients in the training set.

Characteristics	Univariate analysis *p* value	Multivariate analysis	
		HR (95% CI)	*p* value
Age, yrs (＜65)			
≥65	＜0.001	1.175 (1.102–1.254)	＜0.001

Sex (male)			
Female	0.507		

Location (head)			
Body and tail	0.088		
Other	0.608		

Year of diagnosis (2004–2009)			
2010–2015	＜0.001	0.638 (0.597–0.681)	＜0.001

Grade (low grade)			
High grade	＜0.001	1.333 (1.250–1.423)	＜0.001

Examined lymph nodes (＜15)			
≥15	＜0.001	0.800 (0.748–0.856)	＜0.001

Extrapancreatic invasion 8th *T* stage (T1)	＜0.001	1.199 (1.097–1.310)	＜0.001
*T*2	＜0.001	1.340 (1.200–1.473)	＜0.001
*T*3	＜0.001	1.549 (1.394–1.722)	＜0.001

8th *N* stage (N0)			
*N*1	＜0.001	1.336 (1.235–1.445)	＜0.001
*N*2	＜0.001	1.793 (1.639–1.961)	＜0.001

7th AJCC stage (IA)			
IB	＜0.001		
IIA	＜0.001		
IIB	＜0.001		

Abbreviations: HR, hazard ratio; CI, confidence interval.

**Table 3:** Performance evaluation of the 8th AJCC and the novel staging system.

Staging system	AUC for 3-year OS (95% CI)	*p* value	Homogeneity (likelihood ratio *χ*^2^)	Discriminatory ability (linear trend *χ*2)
Training set				
8th AJCC	0.615 (0.598–0.633)		248.6	218.0
Novel	0.642 (0.624–0.659)	<0.001	403.4	335.1

Internal validation set				
8th AJCC	0.603 (0.585–0.621)		182.6	154.7
Novel	0.621 (0.604–0.639)	<0.001	261.9	218.2

External validation set				
8th AJCC	0.596 (0.523–0.670)		15.2	24.6
Novel	0.658 (0.590–0.727)	0.008	33.8	35.7

Higher AUC, discriminatory ability, and homogeneity indicate better performance of the staging system. Abbreviations: AUC, area under the curve. CI, confidence interval.

## Data Availability

The data used to support the findings of this study are available from the corresponding author upon request.
